# A simple seed-piercing transformation protocol for pearl millet and finger millet

**DOI:** 10.1093/aobpla/plaf050

**Published:** 2025-09-11

**Authors:** Mazahar Moin, Wricha Tyagi, Gurijala Sreevarshitha

**Affiliations:** Research Program—Accelerated Crop Improvement (ACI), International Crops Research Institute for the Semi-Arid Tropics (ICRISAT), Patancheru 502324, Telangana, India; Research Program—Accelerated Crop Improvement (ACI), International Crops Research Institute for the Semi-Arid Tropics (ICRISAT), Patancheru 502324, Telangana, India; Research Program—Accelerated Crop Improvement (ACI), International Crops Research Institute for the Semi-Arid Tropics (ICRISAT), Patancheru 502324, Telangana, India

**Keywords:** pearl millet, finger millet, seed-piercing, *Agrobacterium* transformation, gene editing

## Abstract

Pearl millet and finger millet face challenges in adopting transgenic or editing approaches due to their inherent recalcitrance to genetic transformation protocols. To overcome these limitations, the current study aims to streamline the genetic transformation protocol for pearl millet and finger millet. We targeted mature embryos as explants to assess transformation efficiencies, gain in time, and overall feasibility. Notably, a transformation efficiency of 17.74% and 18.79%, for pearl millet and finger millet, respectively, was observed using a method that involved directly piercing the mature seeds at the embryonic region with a needle dipped in *Agrobacterium* suspension, followed by vacuum infiltration. After infection, the seeds were allowed to produce calli and differentiate into shoots and roots, resulting in the development of PCR-positive plants. The induction of infected explants to form calli and subsequent differentiation into shoots and roots, leading to putatively transformed plants, was achieved within 60–66 days. Chi-square analysis of hygromycin selection in T_1_ progeny showed a 3:1 segregation, indicating single-locus inheritance, and PCR of T_1_ plants with *Cas9* and *HptII* primers confirmed intact T-DNA transmission. Furthermore, as a proof-of-concept for transformation leading to gene editing, a grain-specific phospholipase-d delta1 (*PgPLD-delta1-7a*), previously identified in our study, was successfully targeted in pearl millet using the CRISPR/Cas9 approach. This seed-piercing protocol has been successfully evaluated in two genotypes of pearl millet and one genotype of finger millet, resulting in the generation of putative-transformed plants.

## Teaser

Pearl millet and finger millet are important food crops but are notoriously recalcitrant to genetic transformation and gene editing, limiting their improvement through biotechnology. We present a simple and effective seed-piercing protocol that overcomes these challenges. By piercing mature embryos with a needle dipped in Agrobacterium followed by vacuum infiltration, we achieved transformation efficiencies close to 18% in both crops. Within two months, infected seeds regenerated into shoots and roots, producing PCR-confirmed plants that transmitted the introduced DNA to the next generation in a Mendelian 3:1 ratio. As a proof of concept for gene editing, we successfully targeted a grain-specific phospholipase D gene in pearl millet using CRISPR/Cas9. This practical and reproducible approach accelerates the genetic improvement of recalcitrant millets, enhancing crop productivity and resilience.

## Introduction

Transgenic and genome-editing approaches offer promising solutions to address the challenges of enhancing agricultural productivity by transferring potential candidate genes to recipient plant genomes or editing the desired alleles. *Agrobacterium*-mediated genetic transformation is a well-established approach for efficiently transferring T-DNAs carrying different expression cassettes into the plant genome. Over time, this approach has seen various enhancements, commencing with the selection of different explants, infection methodologies, alterations in the composition and strengths of co-cultivation, selection, and regeneration media, and the use of different pathogenic strains of *Agrobacterium* harbouring binary vectors of interest. Similarly, considerable efforts have been dedicated to establishing an efficient *Agrobacterium*-mediated genetic transformation system in cereals. Despite the successful establishment of high-efficiency transformation systems for rice and maize (Jiang et al. 2024, Rengasamy et al. 2024), progress in other cereal crops, particularly pearl millet, has been notably constrained.

Being climate resilient, pearl millet (*Pennisetum glaucum* (L.) R. Br.) and finger millet (*Eleusine coracana* (L.) Gaertn.) hold a crucial position for crop improvement, including diversification. They serve as key players in meeting the nutritional needs and socio-economic welfare of millions worldwide (Satyavathi et al. 2022). The exceptional nutritional properties of pearl millet are leading to its growing recognition as a functional food on a global scale (Samtiya et al. 2023). Although significant advancements have been achieved to enhance productivity through the development of high-yielding cultivars, improved agronomic practices (Satyavathi et al. 2022), and the availability of full-length genome sequence, marker datasets, etc., the full biological potential of pearl millet is yet to be realized. The application of biotechnological tools, including gene editing for functional validation and understanding the genetics underlying key traits, has just started emerging.

Various methods have been employed for the genetic manipulation of plants, such as electroporation, particle bombardment, and the use of *Agrobacterium tumefaciens* (Imani and Kogel 2020). Moreover, a variety of tissue explants, such as immature embryos, mature seeds, shoot apices, floral parts like anthers, and inflorescence, along with calli obtained from these explants, have been determined to be suitable for transformation, contingent upon the particular plant under consideration (Song et al. 2012). Mature embryos served as the optimal starting explants over immature inflorescence or shoot apices in the genetic transformation of cereals due to their ease of acquisition in substantial quantities and maintenance in a viable state for long durations, ensuring their accessibility. The first instances of producing transgenic plants from calli involved co-cultivating mature and immature embryos of rice and maize, respectively, with *Agrobacterium* (Hiei et al. 1994, Ishida et al. 1996). Since then, the transformation protocol, particularly the media composition and the choice of explant, has been refined multiple times. Inducing callus from mature embryos for transformation presents challenges as it is influenced by genotype; however, it is a preferred method as long as effective protocols are in place.

Immature embryos have also been shown to yield transformed plants with different genotypes of cereals (Hiei and Komari 2008, Ishida et al. 2015, Marthe et al. 2015). Nevertheless, the duration required to harvest the immature embryos is significant, and the success of the transformation is also contingent upon the quality of the embryos. The embryogenic calli induced from mature embryos were used as the target explant for transformation in foxtail millet (Santos et al. 2020, Sood et al. 2020). Similarly, a successful tissue culture system was established in 120 sorghum accessions using mature embryos (Li et al. 2017). This led to the effective genetic transformation using embryogenic calli obtained from the mature seeds (Wang et al. 2021). Furthermore, multiple *indica* and *japonica* rice varieties were transformed efficiently using embryogenic calli obtained from mature seeds (Jain et al. 2022). Studies have also demonstrated the generation of somatic embryos, enhanced callus formation, and improved regeneration by overexpressing plant morphogenic genes during the transformation process (Deng et al. 2009, Lowe et al. 2016). The overexpression of the maize morphogenic genes *Baby Boom* (*Bbm*) and *Wuschel2* (*Wus2*) increased the transformation efficiency in maize genotypes that were previously considered recalcitrant (Lowe et al. 2016). This was achieved using embryo slices or leaf segments as the transformation method. The role of these genes in improving transformation efficiency has also been tested in sorghum, sugarcane, and rice (Lowe et al. 2016).

The biolistic method of genetic transformation was among the earliest techniques developed in pearl millet (Goldman et al. 2003). Owing to the disadvantages associated with the biolistic method, an *in vitro* plant regeneration system was established in pearl millet through somatic embryogenesis using seeds, shoot apices, and immature inflorescence as explants, as well as direct shoot organogenesis using shoot apex explants (Jha et al. 2009). The first report on *Agrobacterium*-mediated genetic transformation of pearl millet was published in 2011 (Jha et al. 2011). Consequently, attempts were made to establish the *Agrobacterium*-mediated transformation of pearl millet to generate genetically stable plants using the calli derived from mature embryos as explants (Ramadevi et al. 2014). Both these approaches require additional time, as the 6-week-old calli were employed for transformation. The calli were subsequently subcultured on resting and selection media for another 4 weeks before the induction of shoots and roots (Ramadevi et al. 2014). Additionally, the efficiency reported was also low, ranging from 1.7% to 6.5%.

The genetic transformation of any crop involves two crucial steps: the transfer of T-DNA into the plant genome and the regeneration of plants from transformed cells. These steps must be optimized to ensure an efficient and successful transformation protocol. In many species, the recovery of transformed plants has proven to be challenging due to factors such as the inability of cells undergoing transformation to regenerate (Gelvin 2010). This challenge is also evident when transforming different genotypes of a crop, as some genotypes may be transformed relatively more easily than others. Therefore, the development of robust and reproducible protocols for transforming specific explants across different genetic backgrounds is essential.

In the current study, we present a simple and stable protocol for the effective production of pearl millet as well as finger millet transformed plants using an *Agrobacterium*-mediated transformation method. The efficacy of the protocol outlined in this investigation can be attributed to several key factors. These include the use of mature embryos as explants for *Agrobacterium* infection, vacuum infiltration after infection with *Agrobacterium* suspension, callus formation from infected mature embryos with subsequent selection, formulation of callus induction medium with 2,4-dichlorophenoxyacetic acid (2,4-D) and kinetin, and the continuous application of plant selection antibiotic until rooting and plantlet generation. This transformation protocol was tested on two genotypes (B lines) of pearl millet, ICMB842 and ICMB95444, and one genotype of finger millet (KNE796), yielding *Cas9*-positive plants in all the genotypes tested with 13%–17% efficiency, also suggesting that the protocol is suitable across genotypes and could potentially work in other genotypes and be adapted for the use in other cereals that possess similar embryonic structures. GROWTH-REGULATING FACTOR 4 (GRF4) and its cofactor GRF-INTERACTING FACTOR 1 (GIF1) have been demonstrated to enhance both the efficiency and speed of regeneration in a few cereals, even in the absence of exogenous cytokinins. This chimeric transcription factor also promotes an increase in the number of transformable wheat genotypes that yield fertile transgenic plants without any perturbed phenotype (Debernardi et al. 2020). Our present study involved the use of an in-house vector that incorporates *OsGRF4:GIF1* alongside the *pcoCas9* endonuclease (Yogendra et al. 2025) to effectively transform and generate edits in climate-resilient crops like pearl millet and finger millet. The pcoCas9 variant carries nuclear localization sequences at both termini of the protein to ensure effective nuclear localization and also includes an IV2 intron to mitigate any adverse effects on bacterial growth that could result from possible leaky expression and nuclease activity (Li et al. 2013).

## Materials and methods

### Binary vector preparation for transformation

Previous findings have suggested that the chimaera of the GRF transcription factor and its GIF cofactor enhances the regeneration efficiency in both monocots and dicots (Debernardi et al. 2020). In our study, *GRF4* (*Os02t0701300*) and *GIF1* (*Os03t0733600*) from *Oryza sativa* were cloned to examine their potential in pearl millet and finger millet regeneration or transformation efficiency following a similar strategy as reported for pCAMBIA2300 (Yogendra et al. 2025). In brief, pCAMBIA1302 carrying a green fluorescent marker (mGFP5) was used as a backbone vector for cloning *OsGRF4-GIF1*, which were selected based on their sequence similarity with wheat *GRF4* (*TraesCS6A01G269600*) and wheat *GIF1* (*TraesCS4A01G250600*), respectively. The chimeric gene sequences of *OsGRF4-GIF1* were cloned by combining *OsGRF4* (1182 bp) and *OsGIF1* (684 bp), linked by a four-amino-acid linker comprising four alanine residues. The complete gene cassette encompassing the *OsGRF4-GIF1* chimeric sequence (1878 bp), driven by the rice *Ubiquitin* promoter (1710 bp) and Nos terminator (253 bp), was synthesized by GenScript™. This cassette was inserted using *Sac*I and *Kpn*I restriction sites within the T-DNA region of the binary vector pCAMBIA1302. Subsequently, *pcoCas9* (plant codon-optimized *Cas9*, 4290 bp) amplified from pYPQ150 (Addgene #69301) under *ZmUbi* promoter (2001bp) along with Nos terminator (253 bp) was cloned using *Kpn*I and *Bam*HI (New England Biolabs, USA). The resulting pCAMBIA1302 vector containing *OsGRF4:GIF1* and *pcoCas9*, which will henceforth be referred to as the pCAMBIA-GRF:GIF-Cas9 construct in this manuscript, was employed in the transformation experiments, while the pCAMBIA1302 vector carrying *pcoCas9* without *OsGFR4:GIF1* (designated as pCAMBIA-Cas9) served as the control. The rationale for the inclusion of *pcoCas9* in the vector backbone was to ensure that the same vector, if working, could be used for gene editing by mobilizing the guideRNA (gRNA).

### Seed-piercing method of transformation

Mature seeds of pearl millet genotypes ICMB842 and ICMB95444 (parental lines originating from breeding efforts at ICRISAT, Hyderabad, India) and finger millet genotype KNE796 (genome sequence available) were obtained from the respective breeding teams of ICRISAT, Hyderabad, India. The pearl millet and finger millet seeds were separately surface sterilized with 70% ethanol for 50–60 s and then with 4% H_2_O_2_ (HiMedia Laboratories, India) for 20 min, followed by five washes of 5 min each using autoclaved double-distilled (dd) water. Approximately 50 sterilized seeds, weighing around 450 mg, were placed into a sterile 50 ml Falcon tube. These seeds occupy an area of up to 10 ml within the tube. They were imbibed in 35 ml sterile dd-water, which fills three-quarters of the tube capacity, and subjected to agitation in a rotary shaker at 200 rpm overnight at 28°C to encourage embryonic emergence. This specific volume of water was consistently maintained to ensure that when the tube was positioned horizontally, all seeds remained fully submerged, promoting absorption and bulging of the embryos. After ∼18–20 h, the swollen embryonic portions were used for infection with *Agrobacterium* containing the binary vector.

Single colonies of *A. tumefaciens* strain LBA4404 carrying the binary vectors, pCAMBIA-GRF:GIF-Cas9 and pCAMBIA-Cas9, were incubated overnight in separate 50 ml flasks at 28°C in the 20 ml Yeast Extract Peptone (YEP, HiMedia Laboratories, India) liquid medium with the addition of 25 mg/l rifampicin (Duchefa Biochemie, Netherlands) and 50 mg/l kanamycin (Sigma-Aldrich, USA). About 500 µl of the primary culture was further inoculated in the 20 ml YEP broth containing 25 mg/l rifampicin and 50 mg/l kanamycin and was allowed to grow until the OD_600_ reached 0.6. The *Agrobacterium* culture was then centrifuged at 5000 rpm for 6 min. The pellet was resuspended in 10 ml infection media containing MS (Murashige and Skoog) salts (Murashige and Skoog 1962) and acetosyringone (300 µM). After the overnight bulging of the embryo due to soaking, the seeds were transferred into a sterile 90-mm Petri dish containing the *Agrobacterium* resuspended in the infection medium. This protocol employs a sterile 25-gauge injection needle affixed to a 1-ml syringe for optimal handling. The needle is dipped in *Agrobacterium* suspension and pierced at the base of the embryo, where the radicle will emerge. Because multiple piercings could damage the embryo and impede germination, each attempt involves a single piercing until the needle makes contact with the endosperm, indicated by a noticeable hard surface.

The rationale behind using a needle is to create a fine passage that facilitates the entry of *Agrobacterium* while minimizing damage to the embryo, which is crucial for successful seed germination and callus induction. Consequently, using a fine needle is more suitable for piercing the seeds than other tools like scalpels, which pose a higher risk of damaging the embryo. Because of the small size of embryos, particularly in finger millet, it can be challenging to accurately pierce them with the naked eye, primarily because of visibility constraints. Wearing suitable magnifying eyewear can effectively resolve this challenge. To hold the seed during piercing, 4.5 in. straight, fine-pointed serrated forceps were used (Thermo Fisher Scientific, US). The serrated design of the forceps provided enhanced grip, ensuring a firm hold on the seeds. The seed was held with forceps in the nondominant hand, while piercing was performed using a syringe with a needle held in the dominant hand. After piercing, the seeds were placed in a rotary shaker for 10 min at 100 rpm. The infected seeds were then subjected to vacuum infiltration at 15 mmHg for 15 min in a vacuum desiccator. Following this, the seeds were briefly dried on a sterile Whatman filter paper and then inoculated onto co-cultivation media, where they were kept in the dark for 2–3 days.

After 3 days of co-cultivation (MS along with 2 mg/l 2,4-D, 0.5 mg/l kinetin, and 300 μM acetosyringone) (Supplementary Table S1), the explants were washed in sterile dd-water 2–3 times, briefly blot-dried, and allowed to acclimate in resting media for 10–12 days in the dark at 28°C. The efficiency of callus induction from mature seeds, achieved by the combined application of auxin (2,4-D) and cytokinin (kinetin), ranged between 90% and 100% (Plaza-Wüthrich and Tadele 2012). Hence, these hormones were used for callus induction in our study. The resting media was also composed of MS salts, 2 mg/l 2,4-D, 0.5 mg/l kinetin, 300 µM acetosyringone, and 100 mg/l carbenicillin (HiMedia Laboratories, India). During this phase, the callus initiation began. Some seeds will also undergo germination with the development of a radicle, which was dissected, and the callus segment was subcultured to facilitate the enhanced growth of calli.

After 10–12 days, the embryogenic calli were transferred to selection media whose composition was the same as resting but supplemented with plant selection antibiotic, hygromycin B (Sigma-Aldrich, US) for 7 days in the dark at 28°C. An initial concentration of 3 mg/l of hygromycin was employed for pearl millet, while 15 mg/l was used for finger millet. Because the pCAMBIA1302 vector carries *mGFP5*, after 18–20 days of incubation (initial 12 days in selection-free followed by 7 days in antibiotic selection media), the calli were examined for green fluorescence using a Leica fluorescence microscope (Leica Microsystems Limited, Switzerland) set at 520 nm, and the resulting images were documented using Leica Application Suite version V4.13.0. The callus induction efficiency was calculated as the number of healthy calli after 12 days of culture on resting media to the total number of seeds infected. Subsequently, the calli were transferred to shoot regeneration media, which has MS supplemented with zeatin (1 mg/l) and indole-acetic acid (1 mg/l), along with hygromycin, and incubated in the dark for 12–14 days. The shooting efficiency was determined by the ratio of shoots initiated after 12–14 days of culture in the dark to the total number of healthy calli. The concentration of hygromycin in the shoot induction media was increased to 4 mg/l for pearl millet and 20 mg/l for finger millet. The explants with shoots were subcultured onto the same regeneration media but exposed to light for another 12–14 days. The shoot elongation efficiency was calculated as the percentage of healthy and fully elongated shoots to the total number of shoots obtained. The fully developed shoots originating from the calli were then shifted to rooting media containing MS salts, vitamins, and an appropriate concentration of hygromycin. The rooting efficiency was calculated as the percentage of shoots with fully developed roots to the total number of shoots transferred to the rooting media. The overall transformation efficiency was determined by the ratio of calli induced from infected seeds to the total number of PCR-positive plants obtained through continuous antibiotic selection pressure. Supplementary Table S1 provides a detailed composition of the different media used in this study. After ∼7 days, the emergence of roots was observed, and the plantlets were transferred to Jiffy cups for hardening. After another 7 days of acclimatization, they were transplanted into pots and maintained in the glasshouse at 28° ± 2°C.

### Confirmation of putatively transformed plants

The genomic DNA was isolated using the CTAB (Cetyltrimethyl Ammonium Bromide) method from 100 mg of young leaf tissues obtained from nontransformed and putatively transformed plants from both finger millet and pearl millet separately, after 7–10 days of transfer to soil. The PCR reactions comprised 0.5 μl of dNTP (Deoxynucleotide Triphosphate)s (10 mM), 2 μl of buffer, 0.3 μl of each primer (10 μM), 0.1 μl *Taq* DNA Polymerase (1 U/μl, TaKaRa Bio, Japan), and ∼100 ng of genomic DNA in a 10 μl volume. A 900-bp DNA fragment was amplified using primers that are specific to the *Cas9* gene. Additionally, *HptII* gene primers facilitated the amplification of a 700-bp DNA fragment, and *eIF4* (a housekeeping gene for checking the quality of genomic DNA, Reddy et al. 2015) and *mGFP5* primers were used to amplify fragments of 550 and 450 bp, respectively. Supplementary Table S2 contains the complete list of primer sequences, which includes the gRNAs, flanking primers used for edit confirmation, and primers designed to detect mutations in off-target sites. TaKaRa rTaq (#R001A) was employed for the PCR confirmation of genes located within the T-DNA, while PrimeSTAR GXL DNA Polymerase (#R050A, TaKaRa Bio, Japan) was used for the amplification of amplicons sent for Sanger sequencing reactions. The PCR-amplified products were separated on a 1% agarose gel by electrophoresis at 75 V for 45–60 min.

### Segregation analysis of hygromycin resistance

T_1_ seeds from five PCR-positive T_0_ lines of each construct, pCAMBIA-GRF:GIF-Cas9 and pCAMBIA-Cas9 (ICMB95444 genotype), were surface-sterilized and plated on MS basal medium supplemented with 15 mg/l hygromycin for selection. Seeds from each line were distributed across 10 plates, with 20–23 seeds per plate, maintained at 28°C ± 2°C, 16 h light/8 h dark photoperiod. Germination was scored after 8–10 days, and fully grown seedlings with green leaves were considered resistant, whereas those that did not germinate were recorded as sensitive. Segregation ratios were calculated against the expected 3:1 Mendelian ratio using the Chi-square (*χ*^2^) goodness-of-fit test:


$$\chi^{2}\;=\;\Sigma ({\left(O-E\right)}^{2}/E)$$

where *O* is the observed frequency and *E* is the expected frequency. The degrees of freedom (df) were calculated as *k* − 1, where *k* is the number of categories. Statistical significance was assessed at *P* < .05, with df = 1 and the critical *χ*^2^ value = 3.841.

### Cloning of *PgPLD-delta1-7a* gRNAs and transformation into pearl millet

Previous investigations identified five grain-specific phospholipases through the expression analysis of the phospholipase gene family in pearl millet (Moin et al. 2024). After obtaining *Cas9*- and *HptII*-positive plants from the pCAMBIA-GRF:GIF:Cas9 construct, we wanted to demonstrate the effectiveness of the seed-piercing protocol for generating edits. We targeted one of the grain-specific candidates, *PgPLD-delta1-7a* (PMD7G05807), using CRISPR/Cas9. Two gRNAs, each 20 nucleotides in length and positioned 58 nucleotides apart, targeting the C2 domain, were designed upstream of the GGG and TGG PAM (Protospacer Adjacent Motif) motifs and cloned individually into the pCAMBIA1302 vector containing *OsGRF4:GIF1* and *pcoCas9*. The design of gRNAs involved selecting at least one mismatch in the seed sequence (Chen et al. 2022, Moin et al. 2025). Two sets of primers were designed for the cloning of gRNAs. The first set was for amplifying the *ZmU6* promoter along with the gRNA, achieved by using the *ZmU6* forward primer and the *ZmU6* plus gRNA reverse primer. The second set was designed to amplify the gRNA, scaffold, and poly-T tail, using the gRNA forward primer and the scaffold plus poly-T reverse primer. The amplicons generated from these two PCR reactions were used as templates in the subsequent PCR reaction, which amplified the complete sequence that included *ZmU6*, gRNA, and scaffold. This amplification was performed with the *ZmU6* forward primer and the scaffold plus poly-T reverse primer. The gRNA cassette was incorporated into the binary vector by infusion cloning (TaKaRa Bio, Japan). To confirm the edits, a set of primers flanking both gRNAs was designed to amplify a 640-bp fragment, which was analysed using Sanger sequencing.

Both the gRNA constructs were co-transformed using the seed-piercing method. About 150 µl of the primary culture from each construct was separately inoculated into 50 ml YEP broth supplemented with 25 mg/l rifampicin and 50 mg/l kanamycin, allowing the cultures to grow until the OD600 reached 0.3. Subsequently, the two cultures were mixed in a 1:1 ratio and centrifuged at 5000 rpm for 6 min. The resulting pellet was resuspended in infection media, and seed-piercing transformation was performed as previously described. Three rounds of co-transformations were performed, each involving the infection of 25–30 seeds. Primers flanking the two gRNAs were designed to amplify a 640-bp genomic region encompassing the intended editing sites. The resulting amplicons were subjected to Sanger sequencing, and the sequences obtained were compared with both the control plant transformed with the pCAMBIA-GRF:GIF:Cas9 construct lacking gRNAs and the reference genome sequence obtained from the Novogene Millet Database (Sun et al. 2023) to identify Cas9-induced mutations.

### Off-target mutation analysis in *PgPLD-delta1-7a* edited plant

The analysis of potential off-target sites involved a BLAST search using the 20 bp gRNA and the 3 bp PAM sequence. For each gRNA, three potential off-target sites were selected, and six primer sets were designed to amplify the sequences flanking these potential off-target sites. Subsequently, around 520–570 bp of genomic DNA surrounding the potential off-target regions was amplified in the T_1_ generation. PCR was performed on both the edited plant and the control plant (transformed with pCAMBIA-GRF:GIF:Cas9 construct lacking gRNAs). The resulting PCR products were purified and analysed through Sanger sequencing. Sequence comparison between the edited and control plants was performed to identify any potential off-target mutations.

### Statistical analysis

The mean and standard deviation for independent transformation experiments, performed in two genotypes of pearl millet and one genotype of finger millet using vectors that carry the *OsGRF4:GIF1* and the other that lack it, were used for statistical analysis. One-way ANOVA at *P* < .01 was performed using Sigma Plot v11. For the *χ*^2^ test, statistical significance was assessed at *P* < .05, and the critical *χ*^2^ value is 3.841.

## Results

### Seed-piercing transformation

**Figure 1.**
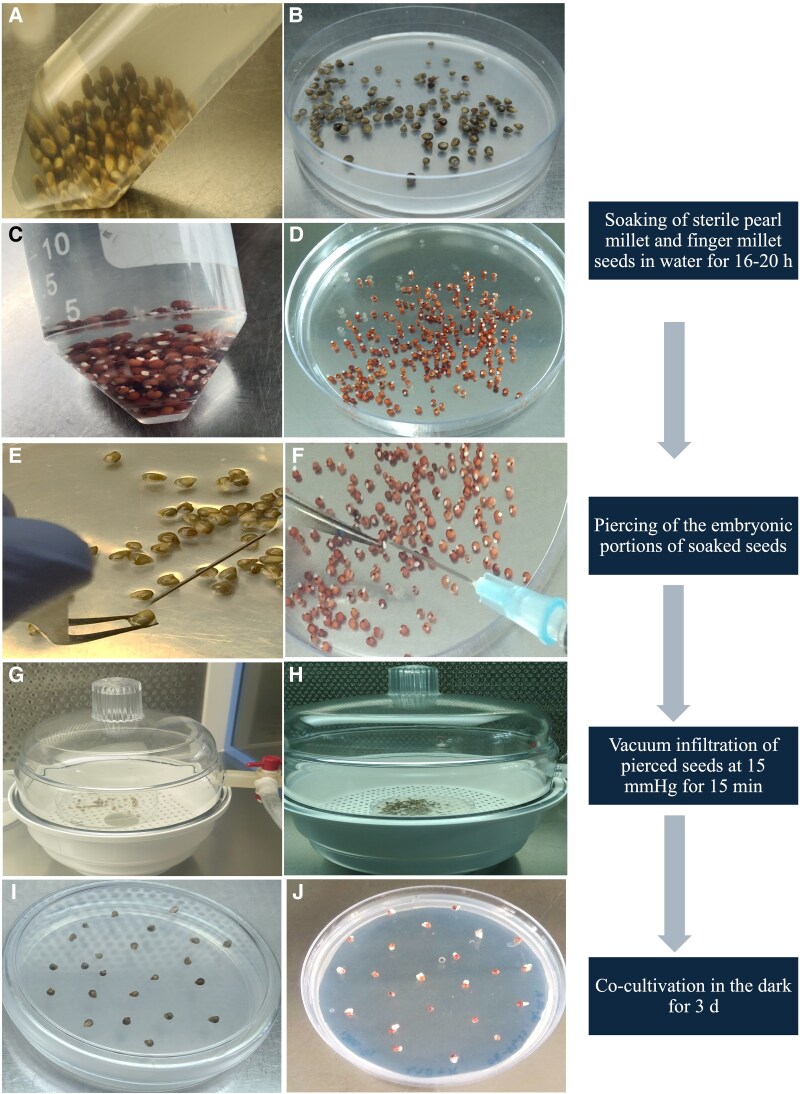
Steps followed for the seed-piercing transformation protocol in pearl millet and finger millet. The pearl millet seeds of ICMB95444 genotype were (A) soaked in water for 18–20 h, (B) subsequently, the soaked seeds were placed in a 90-mm Petri dish containing the *Agrobacterium* suspension. Similarly, the seeds of finger millet belonging to the KNE796 genotype were (C) also soaked for 16–20 h and (D) placed in *Agrobacterium* suspension. The embryonic portion of (E) pearl millet and the (F) finger millet seeds were pierced with a needle that had been dipped in *Agrobacterium* suspension carrying the binary vector, and (G, H) subjected to vacuum infiltration at 15 mmHg for 15 min. The infected (I) pearl millet and (J) finger millet seeds were inoculated on co-cultivation media for 3 days. The flowchart on the right depicts the protocol component of seed-piercing transformation. The protocol commences with the soaking of sterilized seeds in autoclaved water, followed by piercing with a sterile needle dipped in the *Agrobacterium* suspension. The infected seeds were then placed on co-cultivation media for 3 days in the dark.

Previous studies indicated that the chimaera of GRF and GIF can improve regeneration efficiency (Debernardi et al. 2020). The *OsGRF4:GIF1* was cloned into pCAMBIA1302 to investigate its impact on the regeneration or transformation efficiency of pearl millet and finger millet (Supplementary Fig. S1A). The rice GRF4 and GIF1 sequences were selected for cloning, as they showed the highest sequence similarity to their wheat counterparts among the cereal orthologs analysed. Synteny studies show 97% genome collinearity between rice and finger millet (Hittalmani et al. 2017), indicating close evolutionary relatedness. Therefore, the *OsGRF:GIF* sequence is also expected to function effectively in finger millet due to the high conservation of orthologs. Our observations revealed that the *OsGRF4:GIF1* chimaera led to an increase in the size of the callus when compared with the calli transformed with the pCAMBIA-Cas9 vector lacking *OsGRF4:GIF1* (Supplementary Figs S1B and S2). We also assessed the impact of piercing on germination. Our findings indicated no significant difference in germination between pierced and nonpierced seeds; however, the callus induced from pierced seeds was comparatively larger than that from nonpierced seeds (Supplementary Fig. S3A), attributed to the presence of a morphogenic gene (*OsGRF4:GIF1* chimaera) in the construct. If the seeds are viable and stored properly, piercing does not lead to any variation in germination. Hygromycin-sensitivity tests showed complete bleaching of untransformed calli at 3 mg/l, which was selected for effective transformation screening (Supplementary Fig. S3B). One of the advantages of the seed-piercing transformation is that the time required for plant growth, and maintenance to collect explants is saved as the *Agrobacterium* infection targets the embryos of mature seeds (Fig. 1). Unlike other seed-piercing protocols that generate plantlets directly from infected explants (Lin et al. 2009), the formation of calli followed by shoot and root induction is a strategy that was employed in the current study. Also, the addition of antibiotic selection during callus induction, shooting, and rooting ensured that the plants obtained were mostly putative transformants and, thereby, PCR-positive.

**Figure 2.**
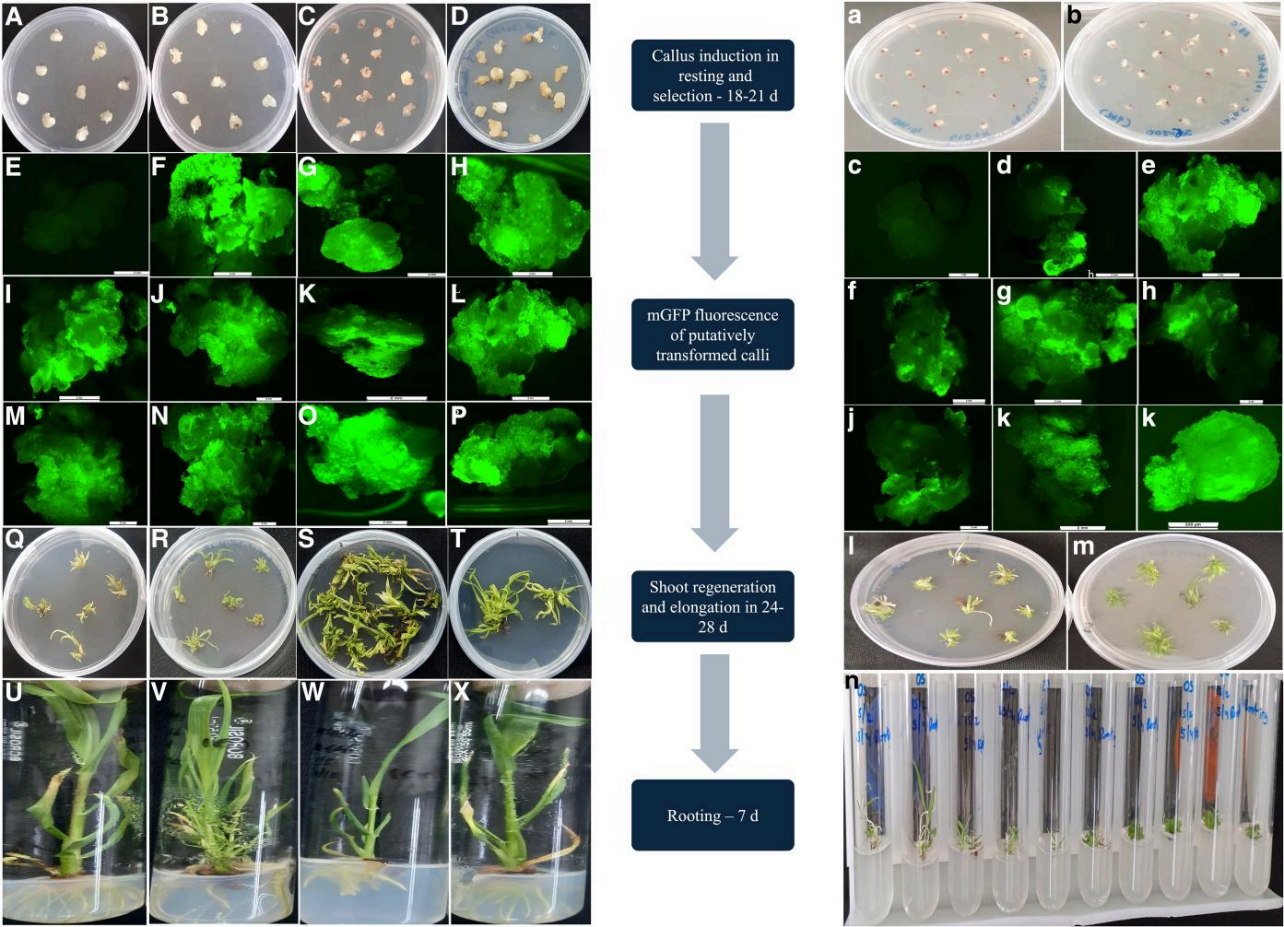
Callus induction, fluorescence imaging, shoot, and root regeneration from *Agrobacterium*-infected pearl millet and finger millet seeds. The illustrations (A–X) in the left panel depict the process of callus induction leading to root development from *Agrobacterium*-infected pearl millet seeds, whereas the visuals (a–n) on the right show corresponding stages from infected finger millet seeds. In the middle, a flow chart is presented, detailing the sequential steps from callus induction to rooting in both crops. After co-cultivation, the pearl millet (A–D) and finger millet (a, b) explants were shifted to resting media for callus induction, which lasted a total of 18–20 days (initially 12 days without antibiotic selection, followed by 7–8 days with hygromycin). The healthy calli that survived in the selection media were analysed for green fluorescence (*mGFP5*) using a Leica fluorescence microscope set at 520 nm. The images were documented with the Leica Application Suite version V4.13.0. Fluorescence was evaluated using nontransformed calli of pearl millet (E) and finger millet (c) as controls to assess the intensity in the corresponding transformed calli (F–P: pearl millet and c–k: finger millet). The fluorescing portions of calli were then transferred to shoot regeneration media containing hygromycin in the dark for 12–14 days, followed by subculturing onto the same regeneration media but under light conditions for another 12–14 days. The fully developed shoots were then transferred to rooting media for 7 days, also in the presence of hygromycin. The duration of this protocol from *Agrobacterium* infection to the successful generation of plantlets spans ∼60–66 days.

The technique of piercing mature embryos of imbibed seeds for *Agrobacterium* transformation has been utilized in rice and other crops (Lin et al. 2009, Zhong et al. 2024). In the present study, this method was applied to pearl millet and finger millet with modifications. During the standardization of the seed-piercing transformation protocol, six rounds of transformation with ICMB842 and three rounds with ICMB95444 genotype using the mature embryos for infection were performed, each carrying around 22–50 seeds for infection. The callus induction efficiency varied between 84% and 92%, demonstrating a higher percentage of callus. This could be attributed to the bulged embryos of mature seeds resulting from soaking along with intact endosperm, which functions as a nutritive tissue at the time of infection with *Agrobacterium* and further growth on callus induction media. This might have aided in the efficient absorption of stored reserves initially from the endosperm and subsequently from the tissue culture media. The fluorescence of the calli was initially observed during 18–20 days after co-cultivation. The calli that displayed bright fluorescence were taken further to the next stages (Fig. 2). Seven days after transfer to the regeneration medium, the fluorescence was re-evaluated. Shoots emerging in the antibiotic selection from these fluorescent portions of the calli were retained subsequently (Supplementary Fig. S4).

The shooting efficiency varied between 41% and 46%. However, some of these shoots did not elongate further. The shoot elongation efficiency was between 62% and 70%. Within 1 week after transfer to half-strength MS, these shoots exhibited root development. The rooting efficiency was 75%–83%. Rooted plantlets were obtained within 60–66 days after infection with *Agrobacterium*. The transformation efficiency was between 12.44% and 17.3% for ICMB842 and 14.26% and 17.74% for ICMB95444 (Table 1). In addition to a moderate increase in transformation efficiency, the time taken for both shoot initiation and elongation is also reduced by 3–4 days in ICMB95444 compared with the ICMB842 genotype of pearl millet.

**Table 1.** Summary of pearl millet and finger millet transformation using the seed-piercing protocol.

Constructs	Genotype	Seeds infected	Callus induction (%)	Shoot induction (%)	Shoot elongation (%)	Root induction (%)	PCR +ve plants	Transformation efficiency (%)
pCAMBIA-GRF:GIF-Cas9	ICMB842	100	86 ± 1.29	42 ± 1.73	27.7 ± 1.25	5 ± 0.81	3 ± 0.5	15.27 ± 1.33
pCAMBIA-Cas9	ICMB842	124	86 ± 4.34	42 ± 3.41	26 ± 3.91	6 ± 0.81	4 ± 0.81	14.87 ± 2.43
pCAMBIA-GRF:GIF-Cas9	ICMB95444	116	83 ± 2.08	40 ± 2.88	34 ± 4.93	6 ± 0.57	4 ± 0.57	16 ± 1.74
pCAMBIA-Cas9	ICMB95444	128	81 ± 2.12	39 ± 4.94	30 ± 8.48	5 ± 0.7	5 ± 1.41	16 ± 1.15
pCAMBIA-GRF:GIF-Cas9	KNE796	108	89 ± 4.16	40 ± 3	29 ± 1.15	6 ± 1.13	4 ± 0.57	16.90 ± 1.89
pCAMBIA-Cas9	KNE796	112	89 ± 3.53	40 ± 0.7	33 ± 4.94	5	4	16 ± 1.35

Two different types of vectors were employed during the transformation: one containing *GRF4:GIF1* and the other lacking this morphogenic gene. The genotypes ICMB842 and ICMB95444 pertain to pearl millet, while KNE796 represents finger millet. The data presented represent the mean ± standard deviation (SD) derived from four independent experiments. The term ‘seeds infected’ refers to the cumulative number of seeds infected across all four independent experiments. The percentages for callus induction, shoot induction, shoot elongation, root induction, and transformation efficiency are calculated as the mean of four independent experiments with SD. One-way ANOVA at *P*  *<* .01 was performed to determine the statistical significance in transformation efficiencies between the constructs carrying *GRF4:GIF1* and those lacking it for a particular genotype. The *P*-value was not <0.01, suggesting that there is no statistical significance in transformation efficiencies.

The seeds of finger millet genotype KNE796 were also subjected to seed-piercing transformation using pCAMBIA:Cas9 and pCAMBIA-GRF:GIF-Cas9 vectors, with each experiment involving 20–30 seeds. The callus induction efficiency achieved from *Agrobacterium*-infected finger millet seeds was 85%–93% (Table 1). These calli also underwent continuous antibiotic selection in callus induction, shooting, and rooting media. The resting media were enriched with MS, phytohormones, and 15 mg/l of hygromycin, with the concentration being increased to 20 mg/l in the regeneration media. As with pearl millet calli, fluorescence in finger millet calli was also detected during 18–20 days of the resting phase. Those calli that exhibited bright fluorescence (Fig. 2) were then advanced to the next stages. The overall transformation efficiency, calculated as the ratio of calli induced from infected seeds to the total number of PCR-positive plants obtained, is 15.1%–18.79% in finger millet transformed with the vector carrying *OsGRF4:GIF1*, whereas the transformation efficiency with the construct lacking this morphogenic gene is in the range of 14.65%–17.35%.

**Figure 3.**
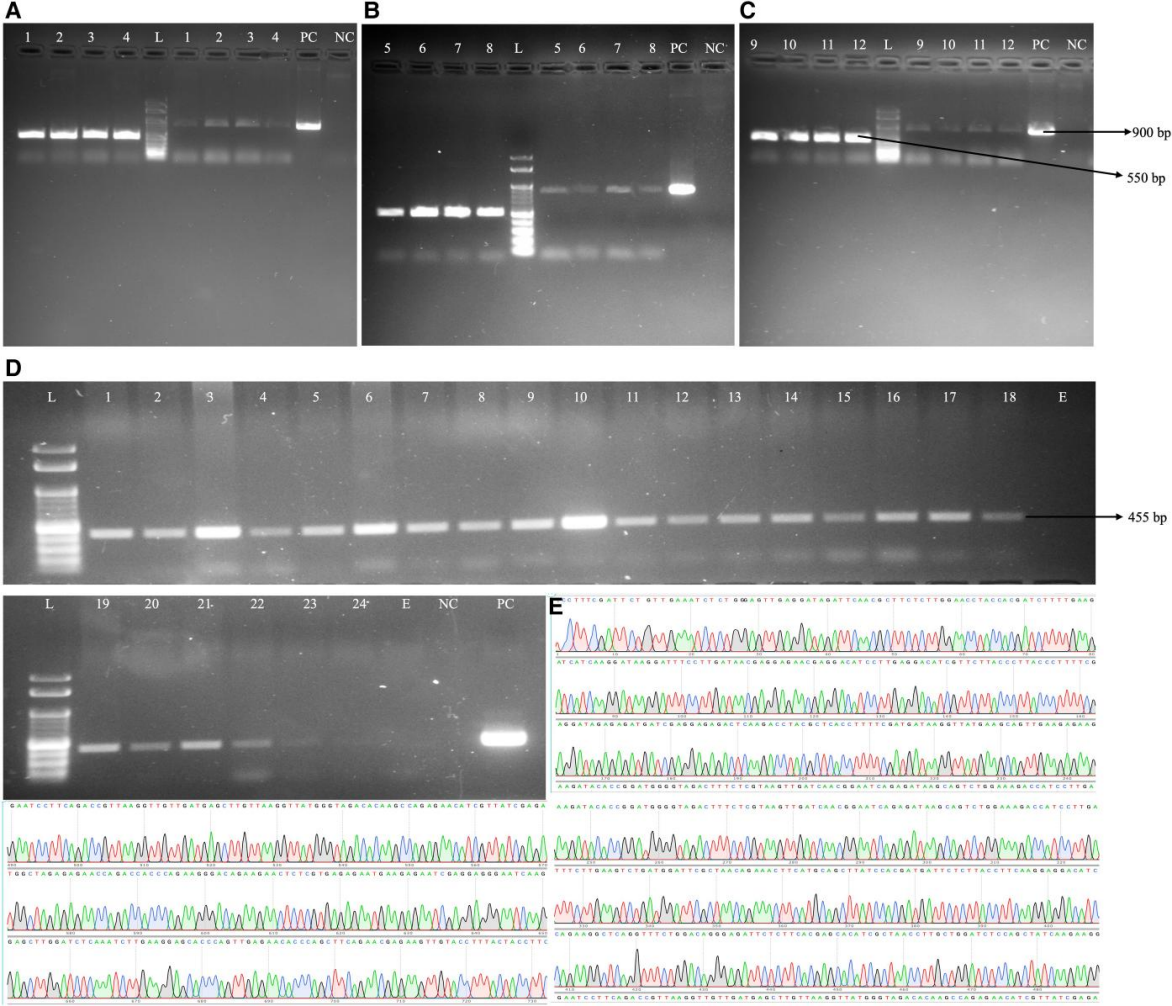
Representative figure showing PCR amplification of putatively transformed plants. PCR amplification was conducted on putatively transformed (A–C**)** pearl millet and (D) finger millet plants. (A–C) PCR amplification of the putatively transformed pearl millet plants with pCAMBIA-GRF:GIF-Cas9 construct, using *eIF4* (550 bp) and *Cas9* (900 bp) primers. Amplification with the housekeeping gene, *eIF4*, served as a quality control for the genomic DNA. (D**)** PCR amplification of finger millet plants putatively transformed with pCAMBIA-Cas9 using *mGFP* primers (455 bp). The labels indicate NC (negative control), PC (positive control), L (100 bp ladder), and 1–24 correspond to genomic DNA samples extracted from the leaves of the putatively transformed plants. The *Cas9* amplicon derived from the putatively transformed pearl millet plants underwent purification and subsequent (E) Sanger sequencing for confirmation.

The PCR analysis was performed on 24 putatively transformed pearl millet plants obtained from the pCAMBIA-GRF:GIF-Cas9 vector, resulting in positive amplification for both *Cas9*- and *HptII*-specific primers (Fig. 3A–C; Supplementary Fig. S5A). A total of 16 plants were obtained from the unaltered pCAMBIA1302 vector, of which 13 were found to be positive upon PCR testing (Table 1). Sixteen putatively transformed plants were produced from the transformation of finger millet using the pCAMBIA-Cas9 vector. Additionally, eight plants were generated using the pCAMBIA-OsGRF:GIF-Cas9 vector. All 24 of these plants were confirmed to be positive for *mGFP* primers (Fig. 3D), and some of these also tested positive for *HptII* primers (Supplementary Fig. S5A). Subsequently, Sanger sequencing with *Cas9* primers confirmed the presence of the target sequences in all samples (Fig. 3E).

### Chi-square segregation analysis of T_1_ progeny and PCR confirmation of T_1_ plants

Screening of 216 T_1_ seeds from ten lines of pCAMBIA-GRF:GIF-Cas9 and pCAMBIA-Cas9 constructs on hygromycin selection medium yielded 148 resistant and 68 sensitive seedlings, closely matching the expected 3:1 ratio. The pooled *χ*^2^ value was 0.002 (*df* = 1, *P* = .964), indicating no significant deviation from Mendelian segregation and suggesting a single-locus transgene insertion. Per-plate analysis revealed that 9 of 10 plates conformed to the expected ratio (*P* > .05), with only one plate showing a minor deviation (*P* = .039, Supplementary Table S3). These results show that hygromycin resistance in T_1_ progeny follows a 3:1 Mendelian ratio, indicating single-locus inheritance and stable transgene transmission.

To evaluate the intact transmission of T-DNA in subsequent generations, 8–10 seeds from 5 *Cas9-* and *HptII*-positive plants derived from pCAMBIA-GRF:GIF-Cas9 vector were germinated, and PCR for *Cas9* and *HptII* were performed in T_1_ (Supplementary Fig. S6). Among the 45 T_1_ plants analysed for PCR, 32 tested positive with both gene-specific primers.

### CRISPR/Cas-mediated *PgPLD-delta1-7a* mutagenesis and off-target mutation analysis

**Figure 4.**
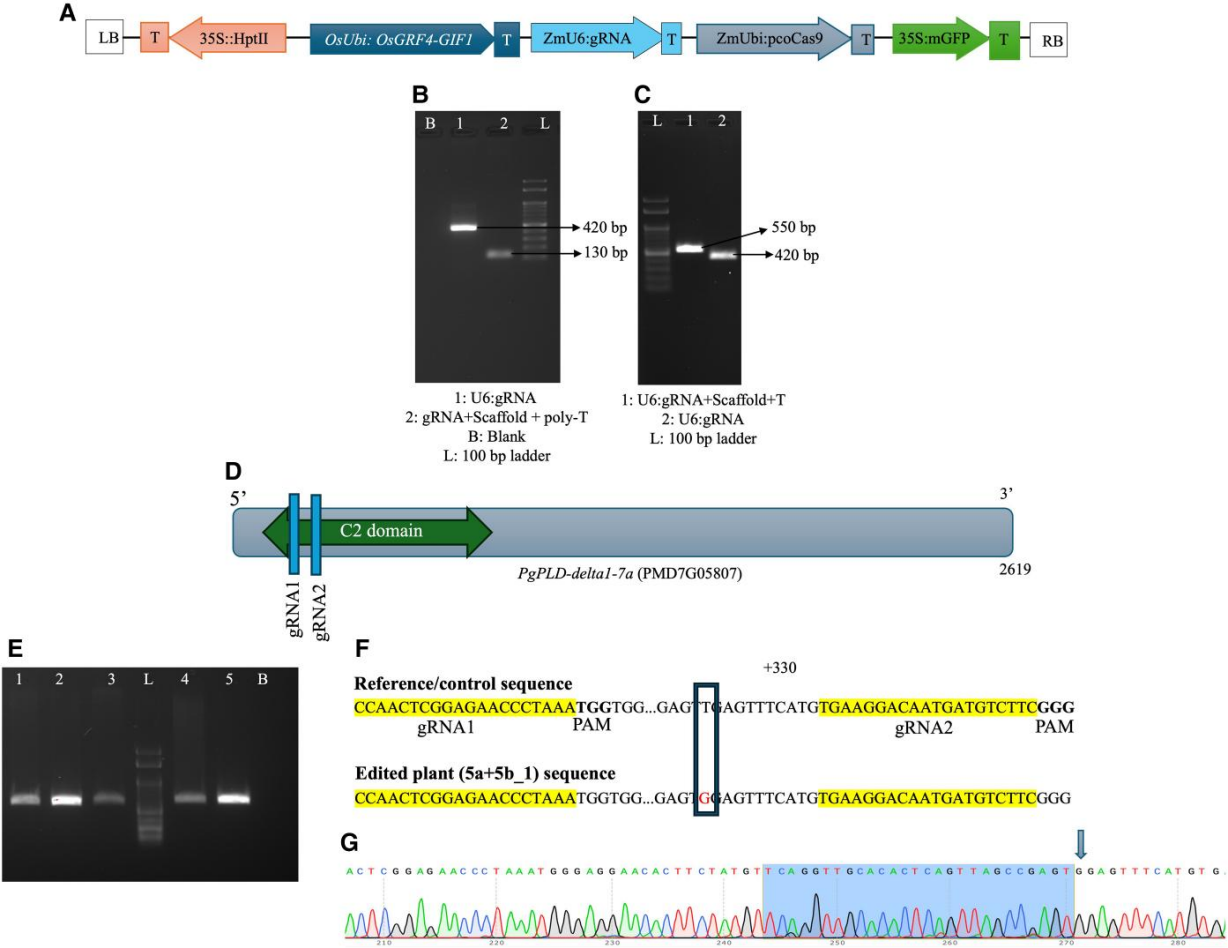
A schematic illustration of the gRNA construct, cloning, and confirmation of the edit in *PgPLD-delta1-7a*. (A) The T-DNA map of pCAMBIA1302 outlines the expression cassettes of the chimeric sequence of *OsGRF4-GIF1*, *pcoCas9*, and gRNA targeting *PgPLD-delta1-7a* driven by the *OsUbi*, *ZmUbi*, and *ZmU6* promoters, respectively. The cloning of gRNA involved the amplification of two fragments. (B) The first amplicon consisted of the *ZmU6* promoter along with the gRNA (combined amplicon size is 420 bp), which was generated using the *ZmU6* forward and *ZmU6* plus gRNA reverse primers. The second fragment (130 bp), comprising the gRNA, gRNA scaffold, and poly-T tail, was amplified using the gRNA forward primer paired with the scaffold plus poly-T reverse primer. (C) These amplicons served as templates for another PCR reaction, which amplified the complete sequence of *ZmU6*, gRNA, scaffold, and poly-T (550 bp) in *cis*. The resulting vector harbouring *OsGRF4: GIF1*, *pcoCas9*, and *PgPLD-delta1-7a* gRNA was employed for *Agrobacterium* mobilization and subsequent plant transformation. (D) The grey box denotes the exon of *PgPLD-delta1-7a*, which spans 2619 bp, while the internal green arrow highlights the conserved C2 domain. The blue vertical lines represent the gRNA target sites at the 5′ end of the exon. (E) The 640 bp amplicon covering both gRNAs underwent Sanger sequencing from five *Cas9*-positive plants (labelled as 1–5, L and B are 100 bp ladder and blank, respectively). (F) The region marked in yellow corresponds to two gRNA sequences, with the bolded sequence indicating the associated PAM (TGG/GGG). The ‘dots’ (…) indicate a similar sequence in the control and edited plant. A thymine-to-guanine transversion was identified in the edited plant (labelled as 5a + 5b_1). (G) Chromatogram of the edited plant 5a + 5b_1 showing the SNP.

Nine plants testing positive for *Cas9* were identified in T_0_. Five of these were subjected to amplification using flanking primers, and the resulting 640 bp amplicon was analysed using Sanger sequencing. While the sequences of the control plant and reference genome are identical for the PMD7G05807, the edited plant exhibited an SNP between the two gRNAs. A thymine-to-guanine base substitution (T/G transversion) was detected in one plant (labelled as 5a + 5b_1) at 11 nucleotides upstream of gRNA2 (Fig. 4D–G). This corresponds to an observed editing efficiency of 20% among the sequenced plants. Ten seeds derived from the single edited T_0_ plant (5a + 5b_1), which resulted from co-transformation involving two *PgPLD-delta1-7a* gRNA constructs, were advanced to the T_1_ generation. One of the 10 T_1_ progeny was *Cas9*-negative and underwent sequencing to reconfirm the presence of the edit using flanking primers. The Cas9-free edited plant was tested for the presence of off-target mutations by PCR amplification of sequences flanking the three potential off-target regions (Supplementary Fig. S7). The PCR-purified amplicons were analysed by Sanger sequencing. The results revealed that the sequences at the off-target sites between the control and the edited plants were identical, suggesting that no mutations were induced in the potential off-target regions of the edited plant.

## Discussion

The application of transgenic or genome editing techniques in functional genomic studies demands the generation of a population of transformed plants with independent T-DNA integration events. A remarkable advancement in plant functional genomics research was attained with the establishment of a simplified and reproducible *in planta* transformation protocol in Arabidopsis. This involves immersing shoots containing inflorescences in *Agrobacterium* suspension, followed by vacuum infiltration and the swift release of the vacuum (Bent 2000). In cereals and other crops, transformation procedures, including *in planta*, have been developed (Lin et al. 2009, Zhong et al. 2024). However, these protocols are restricted to particular genotypes and are not suitable for cultivars that are recalcitrant to tissue culture regeneration. The syntenic relations of pearl millet and finger millet with other cereals suggest that the functional characterization of candidate genes in these two climate-resilient millets will improve their agronomic traits and aid in the comparative genomic studies with the other grass family members.

In the current study, mature embryos of pearl millet and finger millet seeds were infected with *Agrobacterium*, followed by vacuum infiltration. Chi-square analysis confirmed a 3:1 segregation, indicating single-locus inheritance and stable transgene transmission. The intact inheritance of T-DNA to the next generation, demonstrated through PCR targeting *Cas9* and *HptII* genes, reinforces that this protocol is effective for producing stable events. The edit observed in T_0_ in the *PgPLD-delta1-7a-*targeted plant (5a + 5b_1) was confirmed in the T_1_ generation using Sanger sequencing. Additionally, the absence of mutations in the potential off-target regions of this plant was validated in T_1_. This suggests that the current protocol, including the vector used, is viable for generating edits in pearl millet, at least for the two B lines tested in this study. The *PgPLD-delta1-7a* edited plant is currently in the early stages of development and will undergo detailed phenotypic validation, which will be part of our future report. A similar strategy is expected to work in finger millet, as *Cas9*-positive plants were successfully obtained in the genotype tested. The current protocol offers advantages over previous reports that relied on 6-week-old calli for *Agrobacterium*-mediated transformation of pearl millet (Ramadevi et al. 2014). By employing mature embryos as the starting explants for infection, our protocol circumvents the 6-week callus induction period required for infection. We also demonstrated a considerably higher transformation efficiency of 17.74%, in contrast to the previously reported 6.5% (Ramadevi et al. 2014).

Two methods are prevalent for obtaining transgenic plants through *Agrobacterium-*mediated transformation of T-DNA into the host genome. The first method entails the direct generation of the putatively transformed plantlets from the explants, such as shoot apical meristematic (SAM) cells, after *Agrobacterium* infection, leading to the production of chimeric transformed plants (Kesiraju et al. 2021). The second method involves the reprogramming of explants like SAM to produce calli, which subsequently form shoots and roots in the presence of growth regulators (Hiei et al. 2014, Dalton 2020, Sood et al. 2020). This reprogramming of putatively transformed explants to induce calli, producing shoots and roots, usually results in transformed plants showing stable inheritance.

Phenolic compounds are synthesized and stored in plant tissues in response to wounds or stress. These compounds have a dual role of repelling and attracting microorganisms in the vicinity of the plant. Specifically, the low-molecular-weight phenylpropanol derivatives function as chemoattractants for *Agrobacterium* and induce the expression of *vir* genes (Gelvin 2017). An additional factor that promotes *Agrobacterium*-mediated transformation is the stimulation of active cell division after wounding, which facilitates the enhanced binding of *Agrobacterium* to the newly formed cell wall at wound locations and subsequent synthesis of *vir*-inducing compounds (Karami 2008). The efficacy of *Agrobacterium* transformation observed in the present investigation can also be attributed to the spontaneous release of phenolic compounds following injury caused by seed-piercing.

Several studies have examined the effects of different concentrations of phenolics like acetosyringone (ranging from 0 to 400 μM) while optimizing other conditions such as *Agrobacterium* growth (OD_600_ = 0.6–0.8), infection time (10–15 min), and co-cultivation period (1–7 days) (Amoah et al. 2001, Hoque et al. 2005, Ahansal et al. 2022). These findings highlighted that the inclusion of acetosyringone in the infection and co-cultivation media has a substantial impact on transformation efficiency, playing a pivotal role in successful transformation events. The addition of acetosyringone during co-cultivation particularly increases the number of transformed cells in the target tissue in cereals (Ramesh et al. 2004, Ishida et al. 2007, Shrawat et al. 2007, He et al. 2010). Differences in the concentration of acetosyringone required for the successful transformation of cereals might be attributed to differences in *Agrobacterium* infection, the duration of co-cultivation, and, importantly, the competency of the explant (Shrawat et al. 2007). In the current study, a concentration of 300 μM acetosyringone was used, with a total infection period lasting 20–30 min and a co-cultivation duration of 3 days.

The utilization of vacuum treatment for the infection of plant tissues with *Agrobacterium* has proven to be successful in the genetic transformation of cereals (Amoah et al. 2001). It is postulated that the negative-pressure environment created by the vacuum pump promotes effective *Agrobacterium* volatilization, thereby facilitating the entry of *Agrobacterium* into plant cells (Gu et al. 2008). Also, vacuum infiltration entails the expulsion of air from the intercellular spaces due to tissue damage resulting from piercing (Cheng et al. 2004). Subsequently, *Agrobacterial* cells fill the void left by the extracted air upon the release of the vacuum, leading to successful infection. Vacuum infiltration (vacuum treatment coupled with swift release of vacuum) has also been extensively employed in the genetic transformation of Arabidopsis and other crops (Shrawat et al. 2007, Bent 2000). In our experimental setup, a pressure of 15 mmHg for 10 min was applied by the vacuum pump within the vacuum desiccator, resulting in successful transformation events.

The generation of transformed plants in monocots, especially cereals, has predominantly involved inducing callus from immature or mature embryos for plant regeneration. The use of *Agrobacterium*-mediated direct transformation methods has been established for mature seed embryo explants of soybean, cotton, maize, and wheat. For soybean and cotton, explants such as SAM, hypocotyl, and radicles were isolated and directly treated with *Agrobacterium*, followed by the development of transgenic plants (Martinell et al. 2011, Chen et al. 2014 ). Likewise, maize seed embryo explants with plumule were transformed post-isolation, leading to the recovery of transgenic maize plants via multiple bud induction on a high cytokinin medium (Ye et al. 2023). Additionally, wheat mature embryos were also subjected to *Agrobacterium*-mediated transformation, leading to the generation of transgenic plants. Mature embryo explants in these systems do not undergo callus formation, and shoots are directly produced from meristematic cells located around the shoot apical meristem. The chimeric plant regeneration is a major concern in the meristem explant-based transformation systems, as it can affect the determination of the T_0_ plant transgene copy number and decrease the T_1_ transmission rate. Thus, the *Agrobacterium*-mediated infection of a suitable explant, like mature embryos in the present study, and its induction into a callus, and consequent reprogramming of calli to produce putatively transformed plants with stable T-DNA integration, can effectively address the challenges associated with the production of chimeric plantlets.

Callus, a cluster of parenchymal cells, is induced during tissue culture or in response to a wound. In rice, the epidermal cells of the scutellum, essentially the cotyledon of the rice embryo, are capable of regeneration and can initiate the formation of scutellum-derived callus (Bevitori *et al.* 2013). The phytohormone auxin and homologues of genes, including *LEC1 (LEAFY COTYLEDON1)*, which encodes the HAP3 subunit of the CCAAT-box-binding transcription factor, along with the B3-domain genes *LEC2 (LEAFY COTYLEDON2)*, *FUS3* (*FUSCA3*), *ABI3 (ABSCISIC ACID INSENSITIVE 3)*, and the MADS-box transcription factor gene *AGAMOUS-LIKE15* are pivotal in triggering the formation of scutellum-derived callus (Guo et al. 2023). In tissue culture, optimal callus induction was accomplished through the combined application of 2,4-D and kinetin at specific concentrations (Jain et al. 2001). The incorporation of kinetin into 2,4-D-containing media led to an enhancement in the callus induction efficiency (Kaur and Kothari 2004). The current investigation also revealed that the application of 2 mg/l 2,4-D and 0.5 mg/l kinetin was effective in achieving up to 85% callus induction in pearl millet and 92% callus formation in finger millet. Typically, two types of mature embryo-derived calli are suitable for transformation: one is the scutellum-derived callus or primary callus that is cultured for around 2 weeks in the callus-induction medium, and the second is the embryogenic units that originate from the primary callus (Bevitori *et al.* 2013). Calli derived from mature embryos also exhibit significant variations in morphology and texture (Visarada et al. 2002). In rice, at least seven distinct types of embryogenic calli originating from mature seeds, which vary in colour, size, shape, and overall appearance, were identified. These differences are influenced by the media composition, seed quality, and cultivar (Hiei and Komari 2008). In the present study, mature embryos were used for *Agrobacterium* infection, subsequently leading to callus induction. The purpose of inducing callus from infected embryos was to facilitate the regeneration of shoots and roots, ultimately producing plantlets. Therefore, the specific origin of the callus, whether derived from the scutellum or as a secondary embryogenic unit, is not relevant as long as regeneration occurs under selection pressure. Shoots that developed from different calli were regarded as distinct T-DNA events, while multiple shoots arising from a single callus were considered as a single event due to their common origin. Among the two tested genotypes of pearl millet, ICMB95444 demonstrates a significantly faster rate for callus induction, initiation, and elongation of shoots and roots in comparison to ICMB842. We are also currently testing the same transformation protocol for R lines in pearl millet (unpublished data), to have parental material ready with desired alleles.

The current investigation also employed continuous antibiotic selection pressure during the callus-induction, shooting, and rooting stages, which demonstrated efficacy in enhancing the growth of transformed cells. As a result, a majority of the putatively transformed plants that thrived in the glasshouse exhibited amplification of *Cas9*, *mGFP5*, and *HptII* genes, confirming that these plants are potentially transformed. These results are also in agreement with earlier studies that used plant selection antibiotics in the tissue culture medium throughout the organogenesis phases from callus induction to obtaining plantlets (Goldman et al. 2003, Madhavilatha et al. 2006). Using a continuous selection system also effectively prevented any escapes in PCR amplification, as observed in protocols that employed a less stringent selection pressure. In the T_0_ generation, we conducted PCR analysis after the rooting phase, during which the plantlets were transferred to square Jiffy cups for acclimatization. Before this stage, the explant underwent a 6-week selection process in hygromycin (4 mg/l) and carbenicillin (100 mg/l) containing media. In the T_1_, PCR was carried out 15 days after the seeds were germinated in pots. The amplification of *Cas9*, *HptII*, and *mGFP* in the T_0_ and T_1_ generation of transformed plants indicates the integration of T-DNA into the plant genome and its successful inheritance to the next generation, as the persistence of *Agrobacterium* through the antibiotic selection at T_0_ and being transmitted to T_1_ is unlikely.

Transformation procedures in many cereals involve the use of immature embryos that subsequently undergo organogenesis or mature seed-derived embryogenic calli as starting explants for infection with *Agrobacterium* (Hiei et al. 2014, Belide et al. 2017, Nelson-Vasilchik *et al.* 2018). To obtain immature embryos, plants must be cultivated and maintained in a glasshouse until the inflorescence, which typically takes 10–12 weeks, depending on the genotype and species. However, using mature seed-derived embryogenic calli for transformation also presents challenges, as the callus is prone to necrosis and browning when directly exposed to *Agrobacterium*, thereby diminishing its shooting efficiency. By directly employing mature embryos as explants for *Agrobacterium* infection, both the initial plant growth period and the use of callus as an explant can be circumvented. Consequently, transformation using seed-piercing, followed by organogenesis through callus, shooting, and rooting, offers convenience and enhanced flexibility in transgenic plant development.

## Conclusions

This investigation represents one of the few reports on developing a simple, stable, and efficient *Agrobacterium*-mediated transformation protocol for pearl millet and finger millet, which can be effectively applied for gene editing. In this study, we regenerated potentially transformed pearl millet and finger millet plants by targeting the mature embryos for *Agrobacterium* infection. The infected seeds underwent dedifferentiation to produce calli, which were then redifferentiated into shoots and roots while maintaining selection pressure. Piercing followed by vacuum infiltration was essential for effectively delivering T-DNA into the regenerable cells. The current protocol also provides advantages regarding the waiting period for obtaining suitable explants for transformation. Our protocol demonstrated efficiency in terms of time, scalability, consistency, and ease of experimentation. Additionally, this method can be extrapolated to other cereals with similar embryonic structures. Furthermore, this work paves the way for generating edited plants using the protocol and vector optimized in the current study for at least two climate-resilient millets.

## Supplementary Material

plaf050_Supplementary_Data

## Data Availability

The datasets supporting this article are included within this article.
