# Supplementary material for: A simple seed-piercing transformation protocol for pearl millet and finger millet
Source: AoB Plants. 2025 Sep 11;17(5):plaf050. doi: 10.1093/aobpla/plaf050 (PMC12456579; doi:10.1093/aobpla/plaf050)
Supplement: plaf050_Supplementary_Data [file plaf050_supplementary_data.docx]

**Supplementary Figure 1 (SFig. 1)**


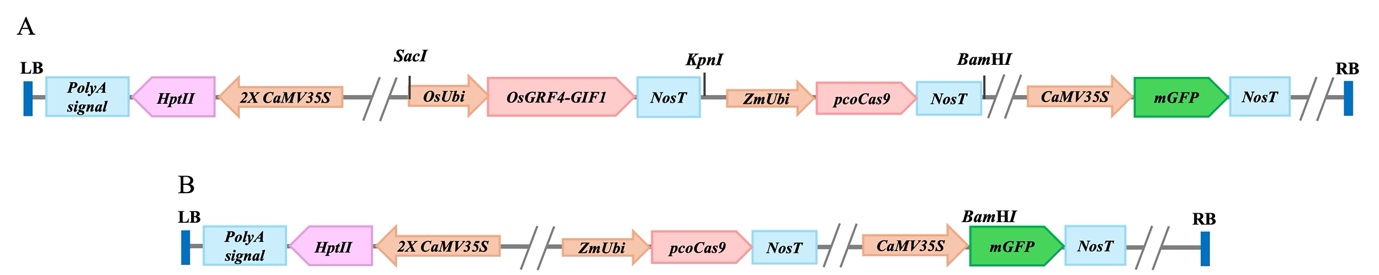


**SFig. 1. A schematic representation of binary vectors used for transformation** (A) The synthesis of a full gene cassette of the chimeric sequence of *OsGRF4-GIF1*, under the regulation of the rice *Ubiquitin* promoter (*OsUbi*) and *Nos* terminator, was carried out by GenScriptTM. This cassette was cloned into the T-DNA region of the binary vector pCAMBIA1302 using *Sac*I and *Kpn*I restriction sites. The expression cassette of the *pcoCas9* under the *ZmUbi* promoter was introduced using *Kpn*I and *BamH*I sites. The resulting pCAMBIA1302 vector harboring *OsGRF4-GIF1* in conjunction with *pcoCas9* was employed in the *Agrobacterium* transformation experiments, while (B) pCAMBIA-Cas9 vector lacking *OsGRF4:GIF1* was used as a vector control.

**Supplementary Figure 2 (SFig. 2)**

**
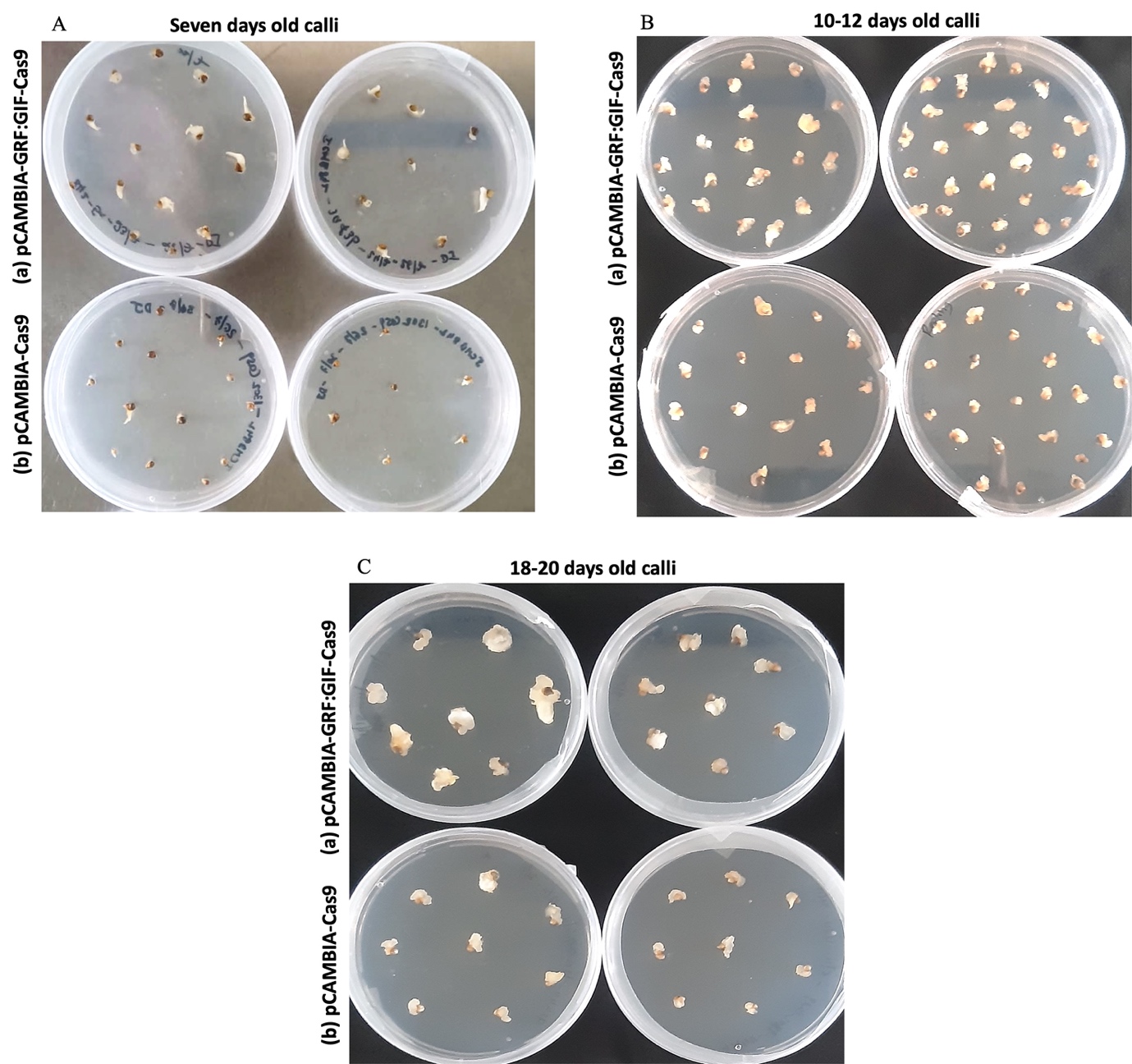
**

**SFig. 2. Comparison of the sizes of calli derived from transformations using vectors with and without morphogenic genes.** The calli of ICMB95444 genotype of pearl millet obtained following seed piercing using pCAMBIA-GRF:GIF-Cas9 (in the upper panel (a) of each figure) and (B) pCMABIA-Cas9 (vector control in the (b) lower panel) constructs. The calli obtained after the transformation of mature embryos with the construct having the morphogenic gene, *OsGFR4:GIF1* exhibited a larger size in comparison to those generated after the transformation with the construct lacking *OsGRF4:GIF1* (pCAMBIA-Cas9).

**Supplementary Figure 3 (SFig. 3)**

**
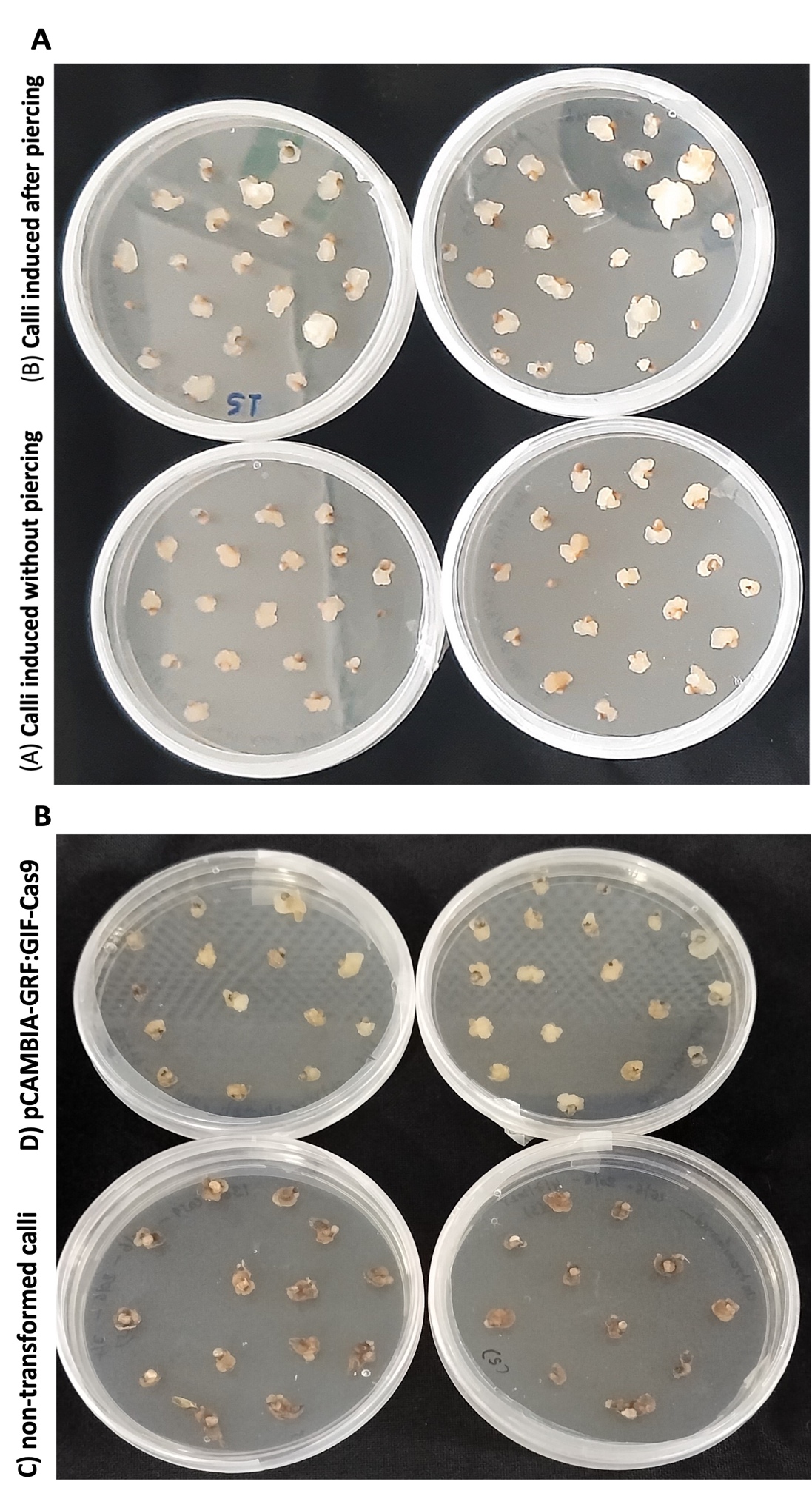
**

**SFig. 3. Effect of (A) seed piercing and (B) hygromycin selection on callus induction and growth in ICMB95444 pearl millet.**

(A) Comparison of callus induction from non-pierced and pierced seeds. Callus was induced from both (A) non-pierced and (B) pierced seeds, showing that piercing does not negatively affect callus induction. However, piercing seeds with a needle dipped in *Agrobacterium* carrying the pCAMBIA-GRF:GIF-Cas9 construct resulted in larger calli, which is attributed to the presence of the morphogenic *OsGRF4:GIF1* cassette.

(B) Hygromycin-sensitivity assay of two-week-old calli. (C) Untransformed calli exposed to 3 mg/L hygromycin underwent complete darkening, whereas (D) calli transformed with pCAMBIA-GRF:GIF-Cas9 continued healthy growth, confirming effective selection.

**Supplementary Figure 4 (SFig. 4)**

**
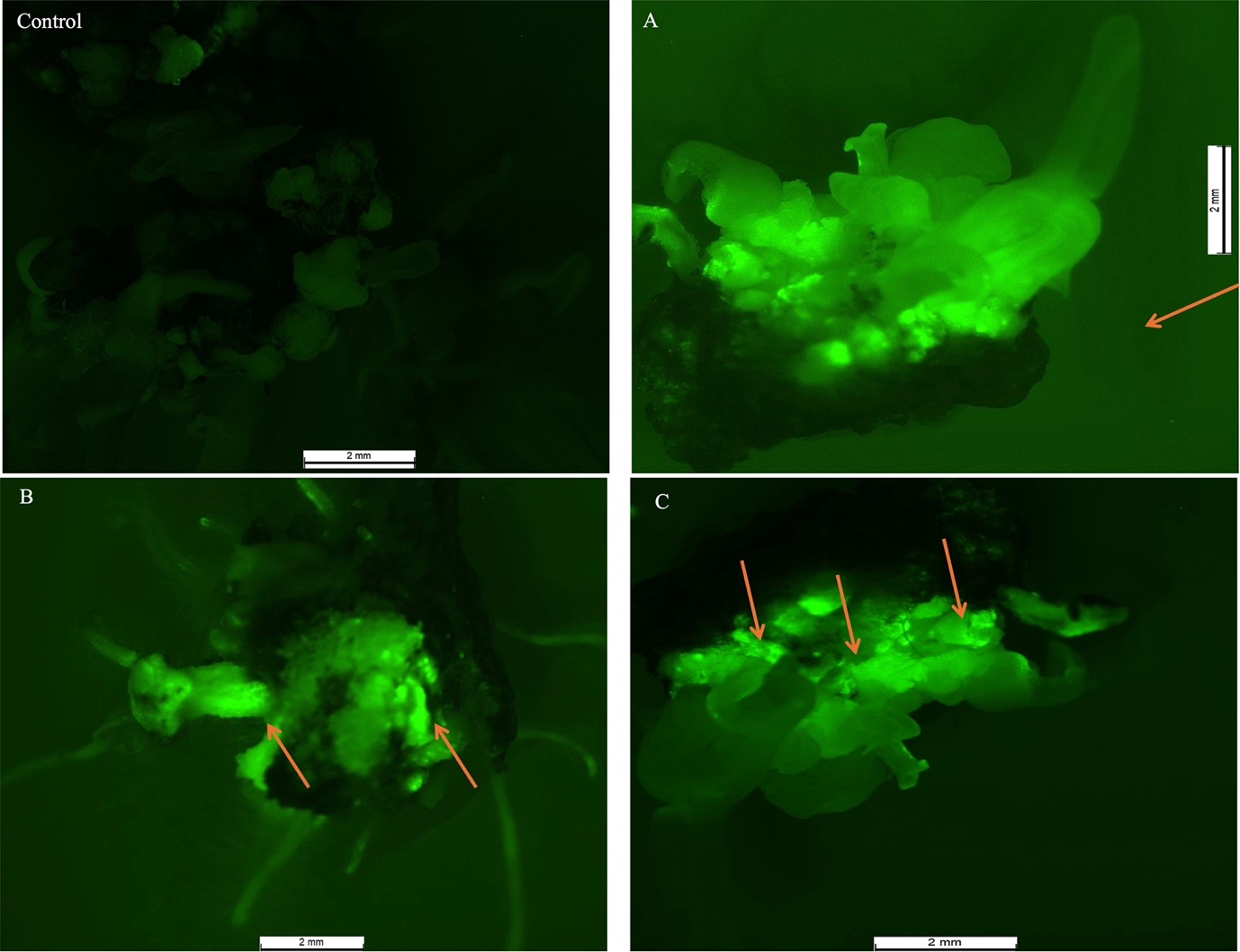
**

**SFig. 4. A representative picture showing fluorescence assessment during regeneration in ICMB95444 genotype of pearl millet.** After two weeks of transfer to regeneration media in the dark, fluorescence levels of transformed and non-transformed calli were once again assessed. Shoots that began to develop from the fluorescing section of the callus were transferred to light and maintained further. Control is the non-transformed calli that are undergoing regeneration. Panels (A-C) depict shoots of the ICMB95444 genotype of pearl millet emerging from the fluorescent regions of the calli. The arrow indicators were used to highlight the specific area on the calli where the shoots were emerging.

**Supplementary Figure 5 (SFig. 5)**


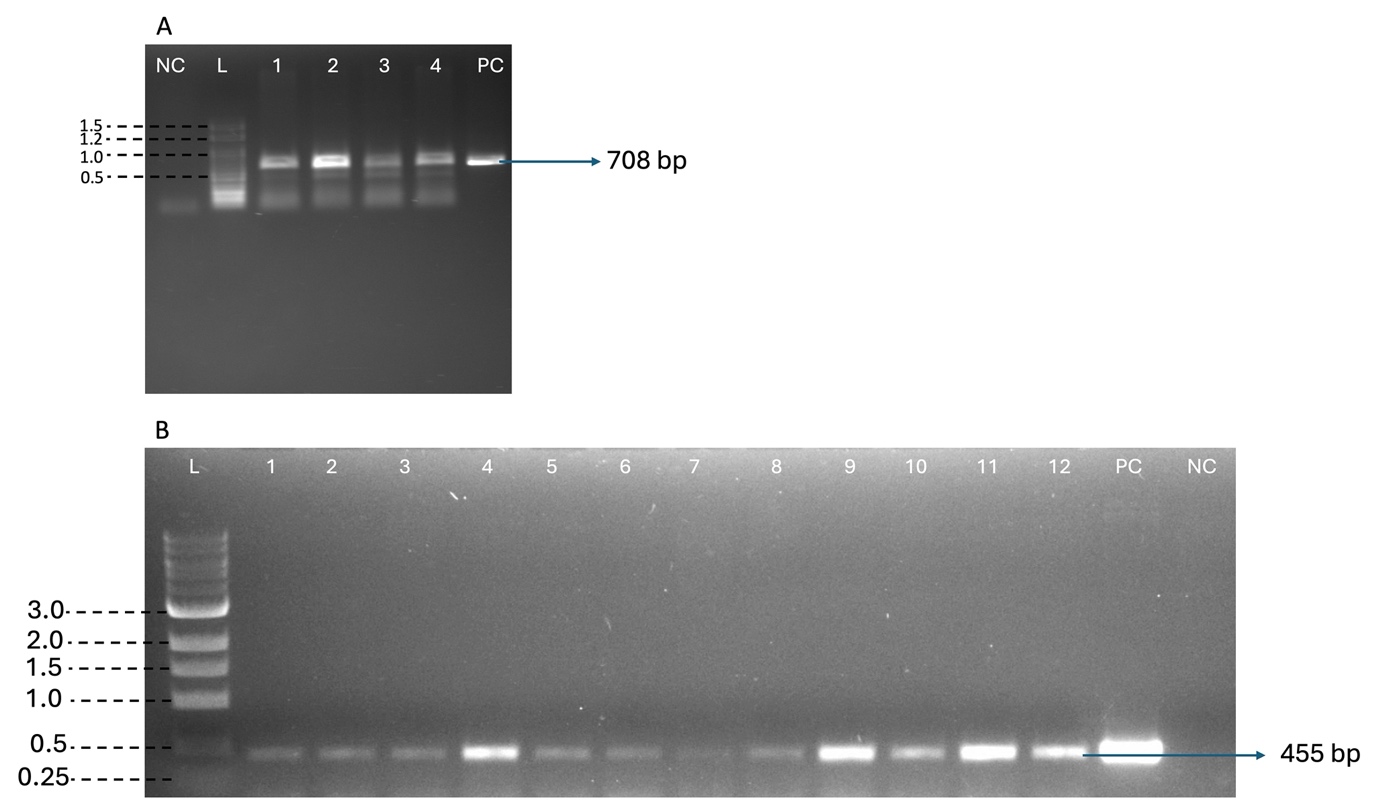


**SFig. 5. A representative PCR picture of finger millet plants.** PCR amplification was performed on putative-transformed finger millet plants obtained with two vectors. (A) Amplification of pCAMBIA-GRF:GIF-Cas9 plants with *HptII* primers (708 bp), and (B) plants obtained with vector control (pCAMBIA-Cas9) were amplified with *mGFP5* primers (455 bp). Labels include NC (Negative control), PC (Positive control), L (100 bp ladder in the first image and 1 kb ladder in the second image), and 1-12, representing genomic DNA samples isolated from the leaves of putatively transformed plants. The ladder is labelled with fragment sizes in kilobases alongside each band.

**Supplementary Figure 6 (SFig. 6).**

**
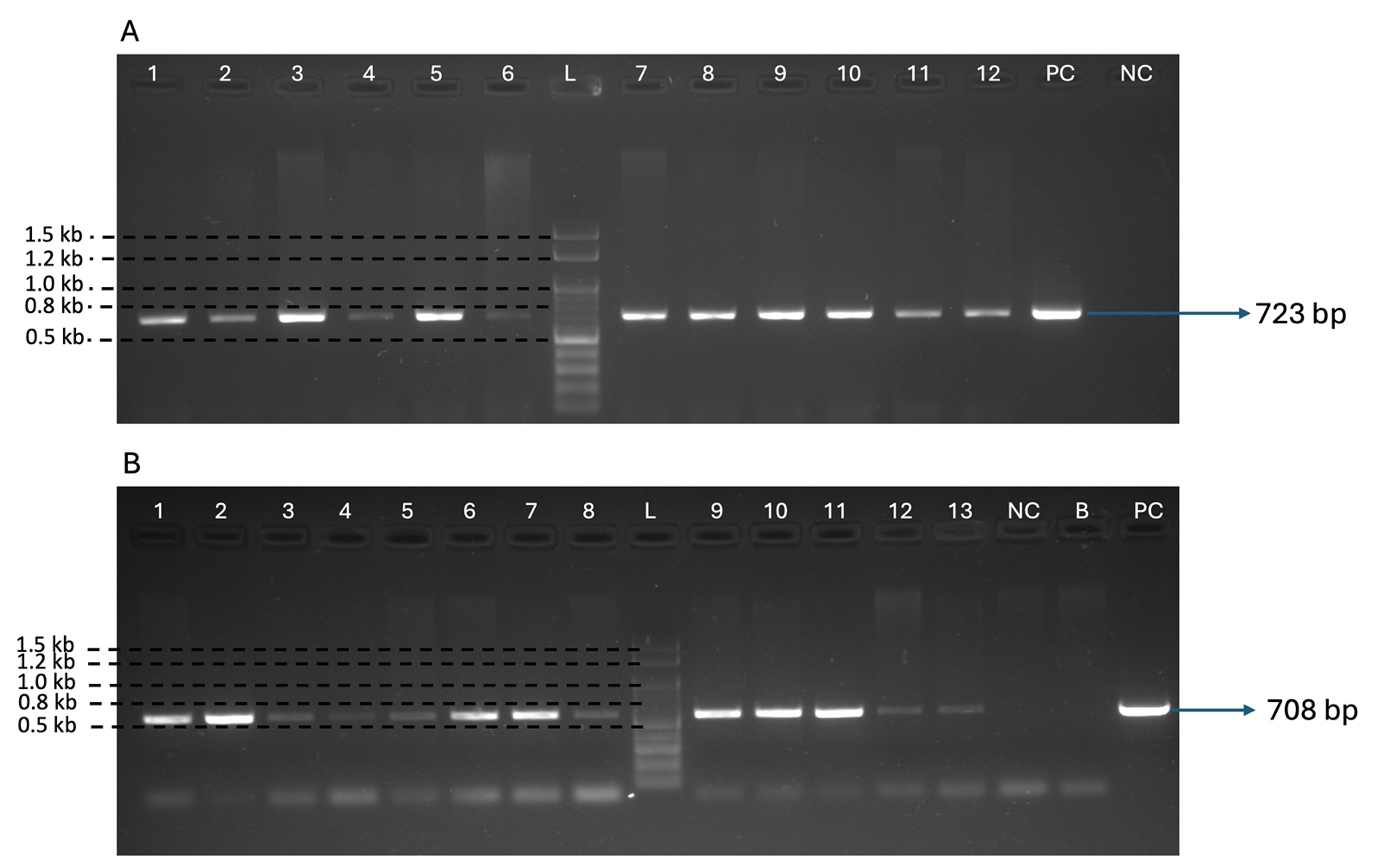
**

**SFig. 6. A representative picture showing PCR amplification of pearl millet plants in T_1_ generation.** The PCR amplification of (A) *Cas9* (723 bp) and (B) *HptII* (708 bp) was conducted in the T_1_ generation of ICMB95444 plants of pearl millet to verify the stable transmission of T-DNA in subsequent generations. Labels include NC (negative control), PC (positive control), L (100 bp ladder), and 1-13, representing genomic DNA samples isolated from the leaves of putatively transformed plants. The ladder is labelled with fragment sizes in kilobases alongside each band.

**Supplementary Figure 7 (SFig. 7)**


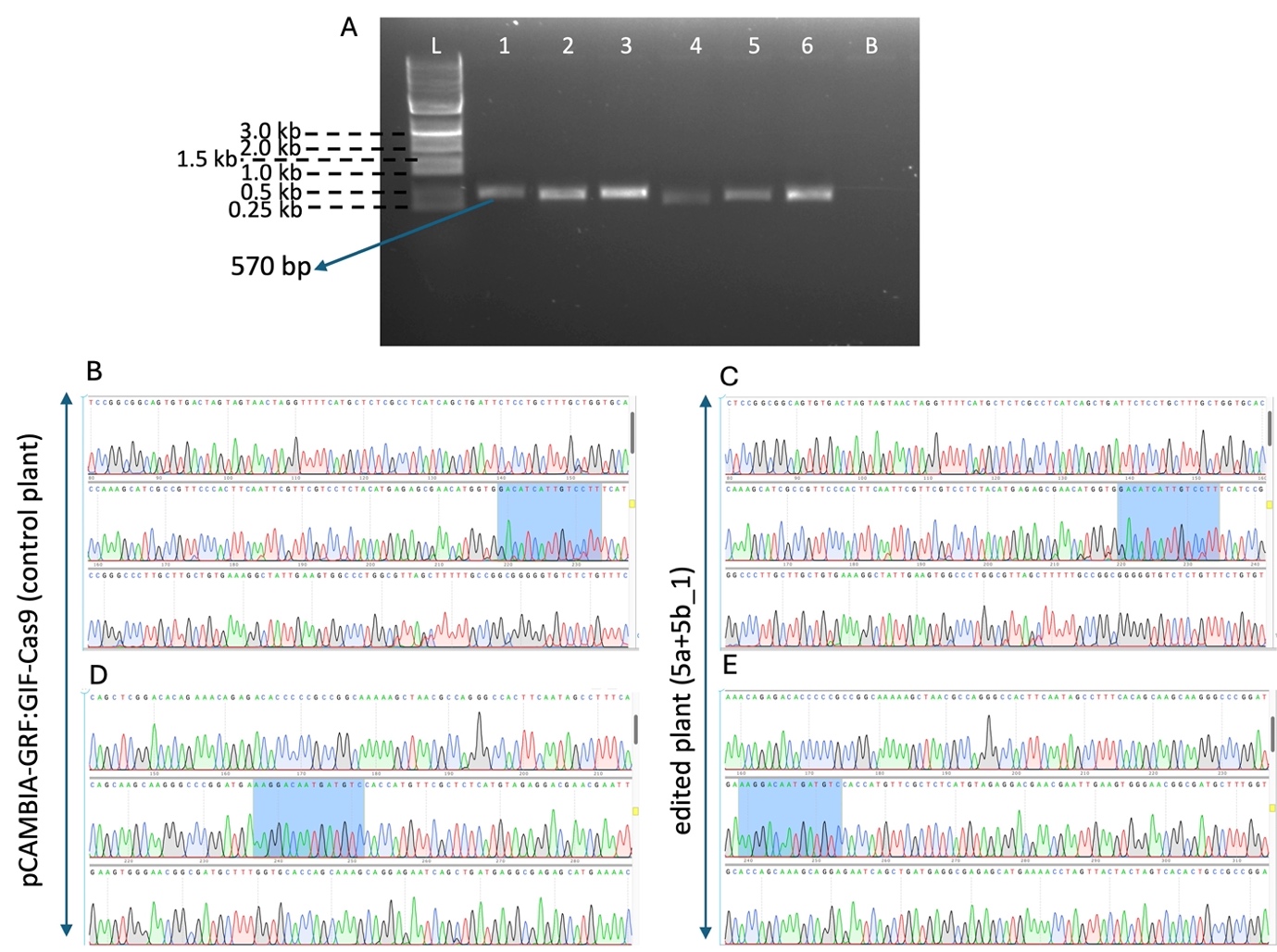


**SFig. 7. Off-target assessment of *PgPLD-delta1-7a*** **(5a+5b_1) edited plant.** Six primer sets were employed to amplify approximately (A) 520-570 bp regions encompassing the potential off-target sites associated with two gRNAs in the T_1_ generation of *Cas9*-free plant that inherited the edit. The resulting amplicons were subsequently purified and analyzed through Sanger sequencing. Labels include L is the 1 kb ladder, 1-6 are PCR amplicons amplified using six different primer sets with genomic DNA isolated from edited plant, B: Blank. The lower panel (B, C, D, and E) illustrates the sequencing results comparing the control plant (left panel; pCAMBIA-GRF:GIF-Cas9 without gRNA) with the edited plant (right panel; 5a+5b_1). Panels (B, and C) depict the sequencing reactions using the forward primers, while panels (D and E) show the reactions with reverse primers. The regions highlighted in blue indicate the potential off-target sites. The ladder is labelled with fragment sizes in kilobases alongside each band.

**Supplementary Table 1 (S1).** Composition of infection, co-cultivation, resting, selection, regeneration and rooting media

| **Components** | **Concentration (working stock)** | **Manufacturer** |
| --- | --- | --- |
| **Infection media (liquid)** | |  |
| MS salts mix | 4.3 g/L | Himedia Laboratories Pvt Ltd, India |
| Myo-inositol | 100 mg/L | Sigma-Aldrich, US |
| Nicotinic acid | 500 mg/L | Duchefa Biochemie, Netherlands |
| Pyridoxine | 500 mg/L | Sigma-Aldrich, US |
| Acetosyringone | 300 µM | Sigma-Aldrich, US |
| Casamino acids | 0.5% (w/v) | Thermo Fisher Scientific, US |
| Sucrose | 68 g/L | Merck Group, Germany |
| Glucose | 36 g/L | Sigma-Aldrich, US |
| **Co-cultivation (solid)** | |  |
| MS salts mix | 4.3 g/L | Himedia Laboratories Pvt Ltd, India |
| Myo-inositol | 100 mg/L | Sigma-Aldrich, US |
| Nicotinic acid | 500 mg/L | Duchefa Biochemie, Netherlands |
| Pyridoxine | 500 mg/L | Sigma-Aldrich, US |
| Thiamine | 1 mg/L | Sigma-Aldrich, US |
| Casamino acids | 0.5% (w/v) | Thermo Fisher Scientific, US |
| 2,4-Dichlorophenoxyacetic acid | 2 mg/L | Himedia Laboratories Pvt Ltd, India |
| Kinetin | 0.5 mg/L | Sigma-Aldrich, US |
| Sucrose | 20 g/L | Merck Group, Germany |
| Glucose | 10 g/L | Sigma-Aldrich, US |
| Agar powder | 8 g/L | Himedia Laboratories Pvt Ltd, India |
| Acetosyringone | 300 µM | Sigma-Aldrich, US |
| Ascorbic acid | 10 mg /L | Sigma-Aldrich, US |
| L-proline | 0.7 g/L | Sigma-Aldrich, US |
| **Resting media (solid)** | |  |
| Co-cultivation media + carbenicillin (100 mg/L) | |  |
| **Selection media (solid)** | |  |
| Resting media + Hygromycin (3-4 mg/L for pearl millet and 15-20 mg/L for finger millet) | |  |
| **Regeneration media (solid)** | |  |
| MS salts mix | 4.3 g/L | Himedia Laboratories Pvt Ltd, India |
| Myo-inositol | 100 mg/L | Sigma-Aldrich, US |
| Nicotinic acid | 500 mg/L | Duchefa Biochemie, Netherlands |
| Pyridoxine | 500 mg/L | Sigma-Aldrich, US |
| Thiamine | 1 mg/L | Sigma-Aldrich, US |
| Casamino acids | 0.5% (w/v) | Thermo Fisher Scientific, US |
| Kinetin | 0.1 mg/L | Sigma-Aldrich, US |
| Cupric sulphate | 1.25 mg/L | Sigma-Aldrich, US |
| Sucrose | 60 g/L | Merck Group, Germany |
| Agar powder | 8 g/L | Himedia Laboratories Pvt Ltd, India |
| Acetosyringone | 300 µM | Sigma-Aldrich, US |
| Ascorbic acid | 10 mg /L | Sigma-Aldrich, US |
| L-proline | 0.5 g/L | Sigma-Aldrich, US |
| Indole acetic acid | 1 mg/L | Himedia Laboratories Pvt Ltd, India |
| Zeatin | 1 mg/L | Himedia Laboratories Pvt Ltd, India |
| Thidiazuron | 1 µM | Sigma-Aldrich, US |
| Abscisic acid | 0.1 mM | Sigma-Aldrich, US |
| Carbenicillin | 100 mg/L | Himedia Laboratories Pvt Ltd, India |
| Hygromycin | 4 mg/L (pearl millet) and 20 mg/L (finger millet) | Sigma-Aldrich, US |
| **Rooting media (solid)** | |  |
| MS salts mix | 4.3 g/L | Himedia Laboratories Pvt Ltd, India |
| Myo-inositol | 100 mg/L | Sigma-Aldrich, US |
| Nicotinic acid | 500 mg/L | Duchefa Biochemie, Netherlands |
| Sucrose | 20 g/L | Merck Group, Germany |
| Phytagel | 3 g/L | Sigma-Aldrich, US |
| Hygromycin | 4 mg/L (pearl millet) and 20 mg/L (finger millet) | Sigma-Aldrich, US |

**Supplementary Table 2 (S2).** Primer sequences used for screening putative-transformed plants

| **Primer Name** | **Sequence (5′-3′)** |
| --- | --- |
| PcoCas9_F | GACCAACAGAAAGGTTACCGT |
| PcoCas9_R | CTTGTTATCGATAGAATCATCCTTC |
| HptII_F | GCCTCCAGAAGAAGATGTTG |
| HptII_R | TGAAAAAGCCTGAACTCACC |
| mGFP_F | GGAGTTGTCCCAATTCTTGT |
| mGFP_R | ATGCCGTTCTTTTGCTTGTC |
| PgEIF4_F | ATCGTGAGCTTTACATCCATCG |
| PgEIF4_R | TATCCCTCAGGATACGGATGTC |
| gRNA1 | CCAACTCGGAGAACCCTAAA |
| gRNA2 | TGAAGGACAATGATGTCTTC |
| 5807-Flank_F | GGGGAAACACTCTTCTGAATCTGG |
| 5807-Flank_R | AGAGTGACCATACCACCCTTCC |
| 5807-gR1_Off1_F | TCACACCTTTTCGCTTACTCTT |
| 5807-gR1_Off1_R | TTTGCAAGGAAAAGAGTACGGT |
| 5807-gR1_Off2_F | GATGGAGCAGACATGAAGACTT |
| 5807-gR1_Off2_R | GCACTTTTACTCACGGATTTGG |
| 5807-gR1_Off3_F | TGGGCAGCTGGGGATGAACA |
| 5807-gR1_Off3_R | TGAGAGCTTCATGGTGAGATCTT |
| 5807-gR2_Off1_F | GGTCTTGTCATGAGGTGCTTAA |
| 5807-gR2_Off1_R | TGAGAGGGAAAGGAGTAGAAGA |
| 5807-gR2_Off2_F | TAGATGGTCCGTACACACACA |
| 5807-gR2_Off2_R | AGCGACGTTCCACATGATTAG |
| 5807-gR2_Off3_F | CTTATCAAGGAAACGGCGATGA |
| 5807-gR2_Off3_R | AATGGCACCAACTGAAGAGAAA |

**Supplementary Table 3 (S3).** Segregation of hygromycin resistance in T₁ pearl millet seeds.

| **Plate** | **Seeds per plate (n)** | **Resistant (Observed)** | **Sensitive (Observed)** | **χ²** | ***p*-value** |
| --- | --- | --- | --- | --- | --- |
| 1 | 20 | 14 | 6 | 0.133 | 0.715 |
| 2 | 22 | 15 | 7 | 0.273 | 0.601 |
| 3 | 23 | 19 | 4 | 0.354 | 0.552 |
| 4 | 20 | 11 | 9 | 4.267 | 0.039 |
| 5 | 22 | 15 | 7 | 0.273 | 0.601 |
| 6 | 20 | 13 | 7 | 0.533 | 0.465 |
| 7 | 22 | 16 | 6 | 0.015 | 0.903 |
| 8 | 23 | 18 | 5 | 0.043 | 0.836 |
| 9 | 21 | 14 | 7 | 0.273 | 0.601 |
| 10 | 20 | 13 | 7 | 0.533 | 0.465 |

Segregation of hygromycin resistance in T₁ seeds obtained from ten T_0_ lines of pCAMBIA-GRF:GIF-Cas9 and pCAMBIA-Cas9 constructs. Observed counts of resistant and sensitive seedlings were compared with the expected 3:1 Mendelian ratio using the Chi-square (χ^2^) goodness-of-fit test with df = 1. p-values were obtained from the χ² distribution to assess significance at p < 0.05.
